# Supplementary material for: Data-independent acquisition method for ubiquitinome analysis reveals regulation of circadian biology
Source: Nat Commun. 2021 Jan 11;12:254. doi: 10.1038/s41467-020-20509-1 (PMC7801436; doi:10.1038/s41467-020-20509-1)
Supplement: Supplementary file 1 — Supplementary Information [file 41467_2020_20509_MOESM1_ESM.pdf]

“Data-independent acquisition method for ubiquitinome analysis reveals regulation of circadian biology”

Hansen *et al.*

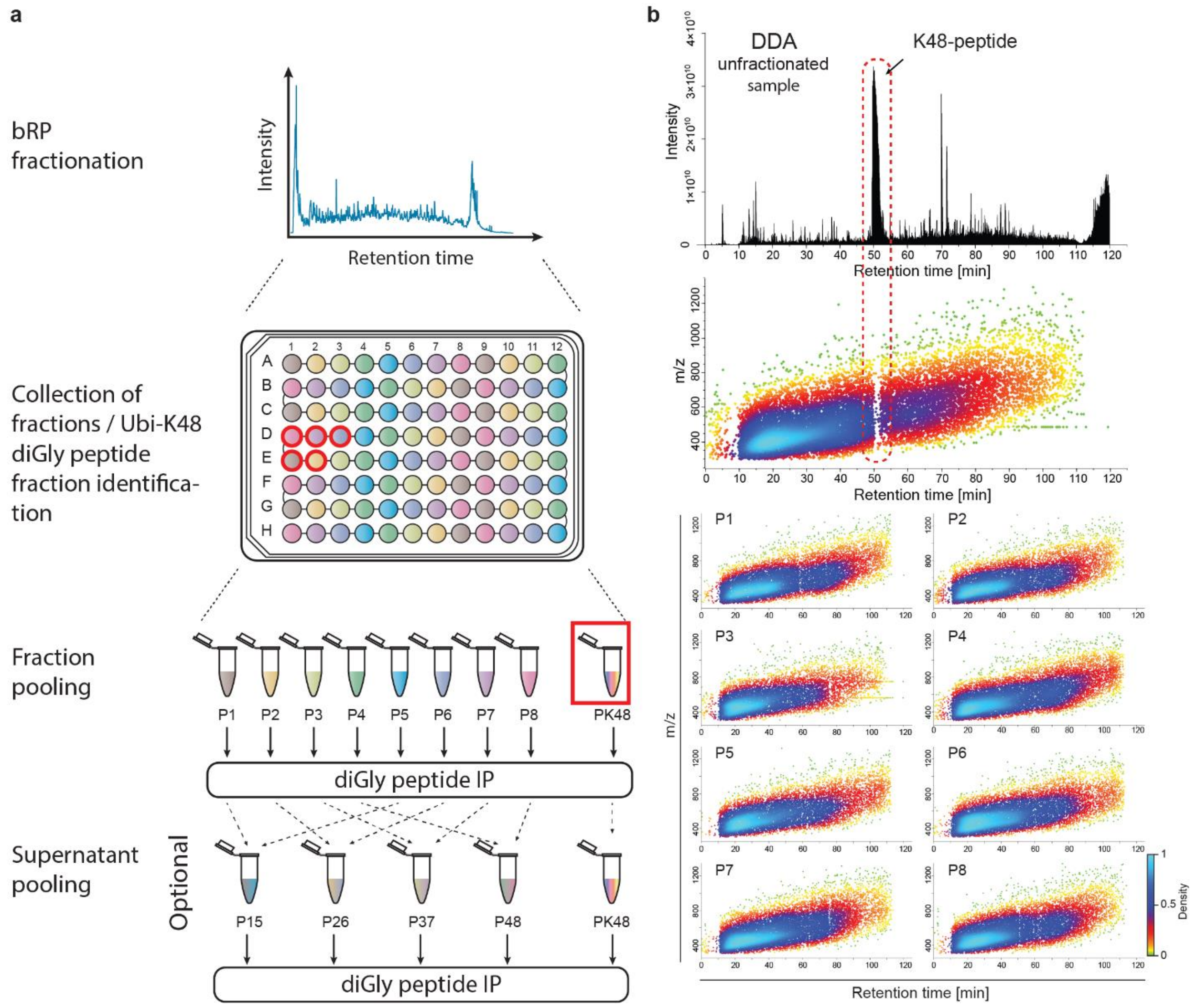

Supplementary Figure 1. Sample fractionation and concatenation scheme

a Workflow for bRP sample fractionation, K48-peptide containing fraction exclusion and concatenation. **b** Characteristic chromatogram and density plot for diGly analysis via DDA (upper panels) and density plots for individual pools (P1-P8). Source data are provided as a Source Data file.

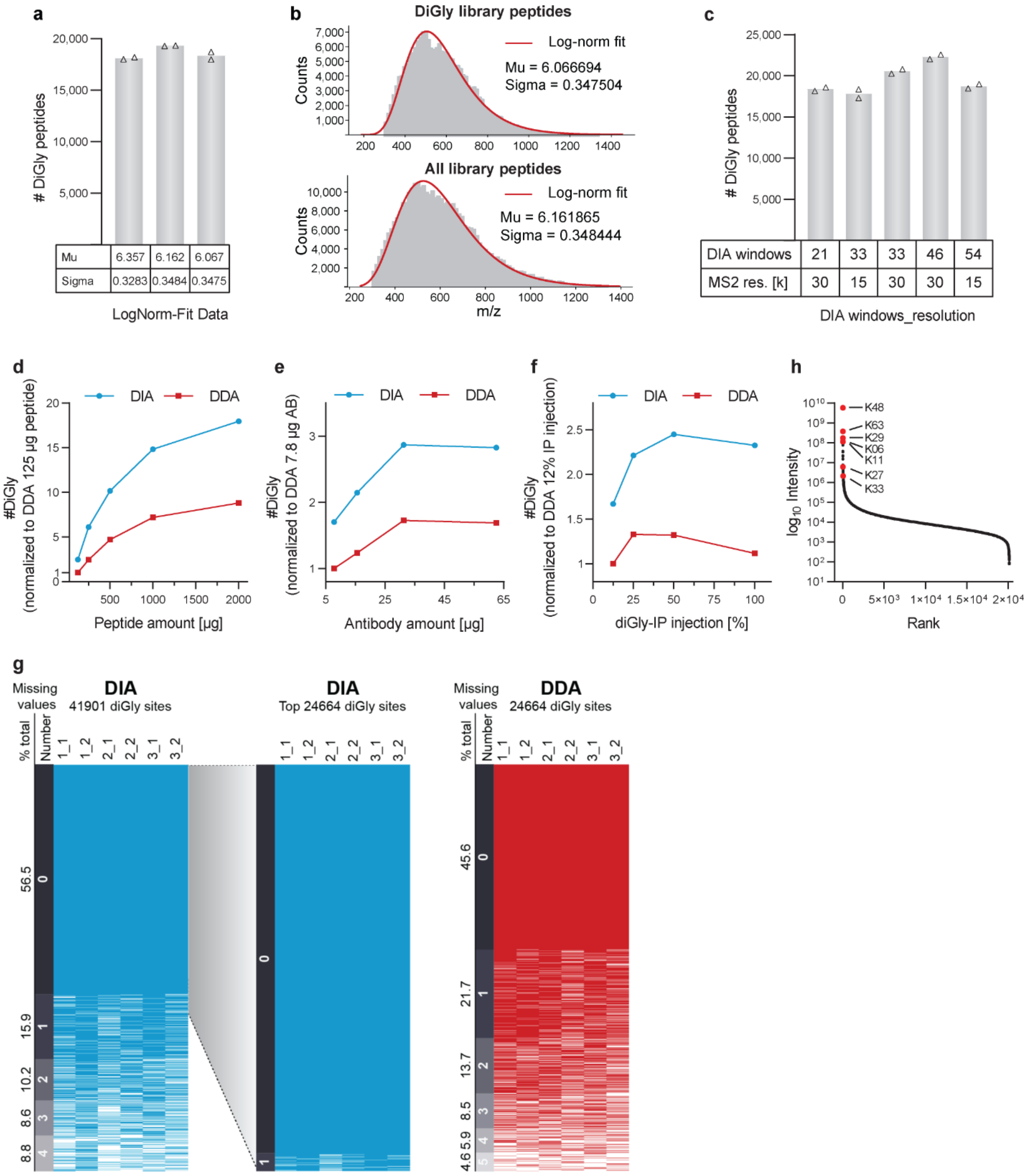

Supplementary Figure 2. DIA method optimization and evaluation

a Number of identified diGly peptides (Mean, n=2) for different m/z peptide distributions (based on different log-Norm fit values: Mu and Sigma). MaxQuant Live default values based on tryptic peptide distribution (left), log-Norm fit values of all identified peptides (middle) and only diGly peptides (right) of HEK293 library peptides. **b** Log-Norm fit curves for diGly (upper panel) and all identified peptides (lower panel) in HEK293 library. **c** DiGly peptide identification (Mean, n=2) for different DIA window numbers and MS2 resolution settings. **d** Relative diGly site identification (Mean, n=2) of different peptide starting amounts using 31.25 µg antibody for enrichment. **e** Same as **d** with constant peptide input (1 mg) and varying antibody amounts. **f** Same as **d** with constant peptide (1 mg) and antibody amount (31.25 µg) with varying sample injection amounts **g** Missing intensity values between analytical and workflow replicates for DIA (blue) and DDA (red) diGly measurements. **h** Dynamic range of diGly peptides. Red dots highlight ubiquitin chain linkage derived diGly peptides. Source data are provided as a Source Data file.

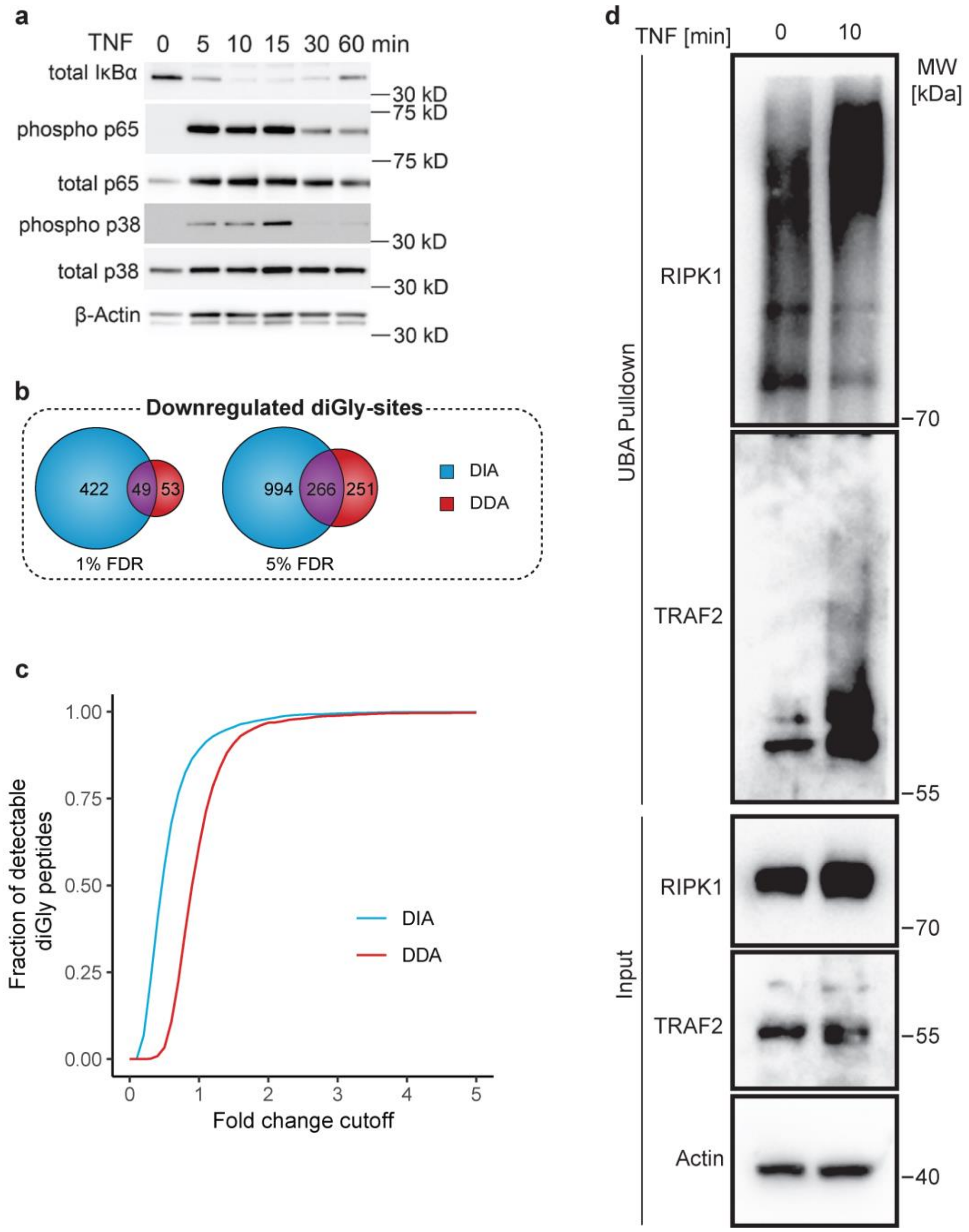

Supplementary Figure 3. TNF-regulated ubiquitinome analysis

a Validation of induced TNF signaling upon TNF stimulation (Top - IκBα, Phospho-NF-κB p65 (Ser536), NF-κB p65, Phospho-p38 MAPK (Thr180/Tyr182), p38 MAPK, β-Actin – bottom; experiment was performed twice with similar results obtained) . U2OS cells were stimulated with 100 ng/ml TNF for the indicated times. **b** Overlaps of significantly upregulated diGly sites between DIA and DDA measurements for 1% and 5% FDR cutoffs (FDR controlled, two-sided t-test, randomizations=250; s0=0.1), respectively (n=6, three workflow replicates measured in analytical duplicates). **c** Power analysis showing fraction of peptides with detectable differences across increasing fold-change cutoffs (power=0.8; sample size=6; significance threshold=1%). **d** RIPK1 and TRAF2 abundance was were assessed by western blot before (Input) and after UBA pulldowns of TNF treated and untreated U2OS cells (experiment was performed twice with similar results obtained). Source data are provided as a Source Data file.

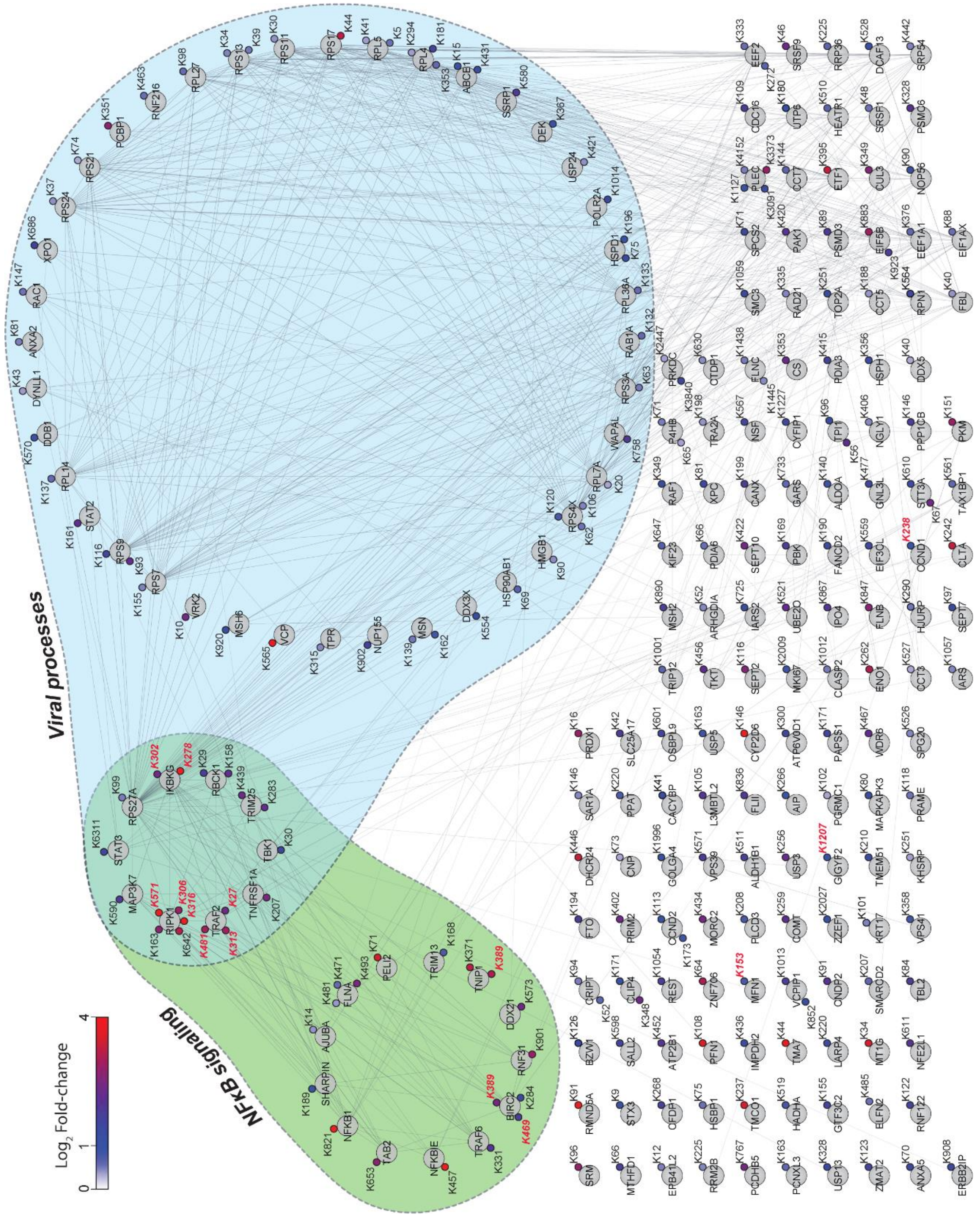

Supplementary Figure 4. Network of TNF-regulated ubiquitinated proteins

Cytoscape network of diGly proteins with significantly upregulated diGly sites (DIA, 5% FDR). Green and blue areas mark ubiquitinated proteins associated with NFκB signaling (GO 0043122 and GO 0051092) and viral processes (GO 0016032). Upregulated diGly sites also identified by DDA (5% FDR) are shown with red font. Source data are provided as a Source Data file.

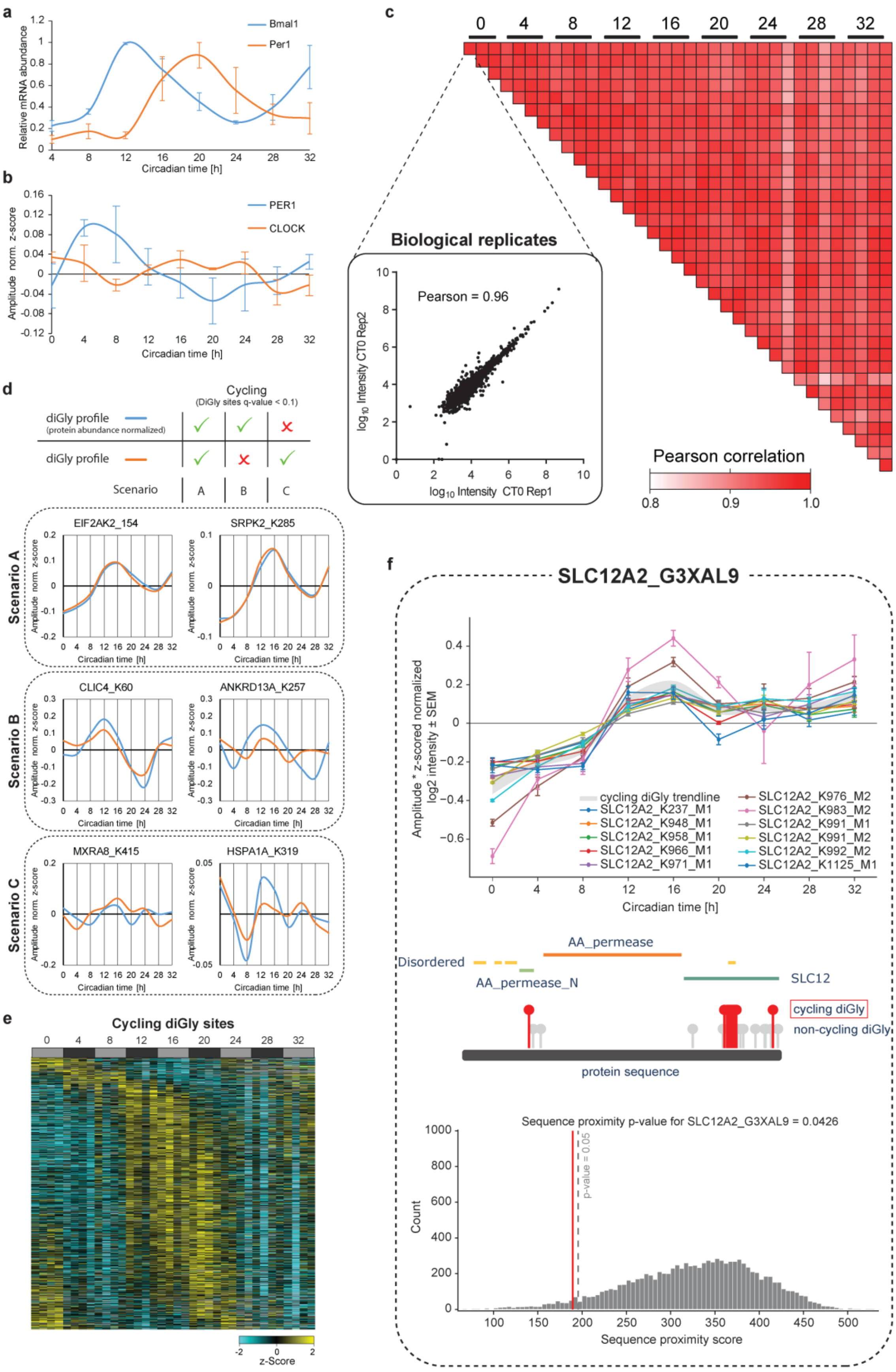
a
Bmal1
Per1
Relative mRNA abundance
Circadian time [h]
b
PER1
CLOCK
Amplitude norm. z-score
Circadian time [h]
c
Biological replicates
Pearson = 0.96
log10 Intensity CT0 Rep2
log10 Intensity CT0 Rep1
Pearson correlation
d
Cycling
(DiGly sites q-value < 0.1)
diGly profile
(protein abundance normalized)
diGly profile
Scenario
A
B
C
Scenario A
EIF2AK2_154
SRPK2_K285
Scenario B
CLIC4_K60
ANKRD13A_K257
Scenario C
MXRA8_K415
HSPA1A_K319
e
Cycling diGly sites
z-Score
f
SLC12A2_G3XAL9
Amplitude * z-scored normalized log2 intensity ± SEM
cycling diGly trendline
SLC12A2_K237_M1
SLC12A2_K948_M1
SLC12A2_K958_M1
SLC12A2_K966_M1
SLC12A2_K971_M1
SLC12A2_K976_M2
SLC12A2_K983_M2
SLC12A2_K991_M1
SLC12A2_K991_M2
SLC12A2_K992_M2
SLC12A2_K1125_M1
Circadian time [h]
AA_permease
Disordered
AA_permease_N
SLC12
cycling diGly
non-cycling diGly
protein sequence
Sequence proximity p-value for SLC12A2_G3XAL9 = 0.0426
p-value = 0.05
Count
Sequence proximity score

Supplementary Figure 5. Circadian regulation of the ubiquitinome

a Average mRNA abundances (±SEM) of Bmal1 (blue) and Per1 (orange) relative to Gapdh control in each time point (n=3 biologically independent experiments). **b** Protein profiles (z-scored and amplitude normalized) of core clock proteins Per1 and CLOCK (n=4 biologically independent experiments). **c** DiGly proteome Pearson correlation matrix. Scatter plot shows the correlation of a representative biological replicate **d** Possible outcomes for protein abundance normalization of diGly site profiles (upper panel). Median amplitude normalized z-score values for diGly profiles with and without protein abundance normalization are shown in scenario-specific example plots (lower panels) with blue and orange lines, respectively. **e** Heat map of intensities ($\log_2$ z-score normalized) of ubiquitination sites (rows) over time (columns) ordered by phase of oscillation. **f** An example of the proximity analysis of cycling ubiquitin clusters (http://cyclingubi.biochem.mpg.de). Cycling sites (q-value <0.1, ±SEM, n=4 biologically independent experiments for each time point) (top) and their location in the protein sequence along with domain annotation (middle) and proximity score (average distance, p-value <0.1) (bottom) are displayed for SLC12A2 (p-value=0.0426). Source data are provided as a Source Data file.
