# Supplementary material for: Data-independent acquisition method for ubiquitinome analysis reveals regulation of circadian biology
Source: Nat Commun. 2021 Jan 11;12:254. doi: 10.1038/s41467-020-20509-1 (PMC7801436; doi:10.1038/s41467-020-20509-1)
Supplement: Supplementary file 3 — Description of Additional Supplementary Files [file 41467_2020_20509_MOESM3_ESM.docx]

**Description of Additional Supplementary Files**

File Name: Supplementary Data 1

Description: DIA method optimization

File Name: Supplementary Data 2

Description: DIA method evaluation

File Name: Supplementary Data 3

Description: Benchmarking analysis comparing DIA and DDA

File Name: Supplementary Data 4

Description: Comparison of DDA and DIA in a biological setting – analysis of TNF signaling

File Name: Supplementary Data 5

Description: Application of DIA for the ubiquitinome analysis of the circadian cycle

File Name: Supplementary Data 6

Description: DIA window scheme

File Name: Supplementary Data 7

Description: PRIDE upload overview

File Name: Source Data

Description: Raw data underlying display data
