# Supplementary material for: Data-independent acquisition method for ubiquitinome analysis reveals regulation of circadian biology
Source: Nat Commun. 2021 Jan 11;12:254. doi: 10.1038/s41467-020-20509-1 (PMC7801436; doi:10.1038/s41467-020-20509-1)
Supplement: Supplementary file 11 — Reporting Summary [file 41467_2020_20509_MOESM11_ESM.pdf]

nature research

Corresponding author(s): Matthias Mann and Ozge Karayel

Last updated by author(s): Nov 20, 2020

# Reporting Summary

Nature Research wishes to improve the reproducibility of the work that we publish. This form provides structure for consistency and transparency in reporting. For further information on Nature Research policies, see our Editorial Policies and the Editorial Policy Checklist.

## Statistics

For all statistical analyses, confirm that the following items are present in the figure legend, table legend, main text, or Methods section.

| n/a | Confirmed | |
|---|---|---|
| ☐ | ☒ | The exact sample size (*n*) for each experimental group/condition, given as a discrete number and unit of measurement |
| ☐ | ☒ | A statement on whether measurements were taken from distinct samples or whether the same sample was measured repeatedly |
| ☐ | ☒ | The statistical test(s) used AND whether they are one- or two-sided<br>*Only common tests should be described solely by name; describe more complex techniques in the Methods section.* |
| ☒ | ☐ | A description of all covariates tested |
| ☐ | ☒ | A description of any assumptions or corrections, such as tests of normality and adjustment for multiple comparisons |
| ☐ | ☒ | A full description of the statistical parameters including central tendency (e.g. means) or other basic estimates (e.g. regression coefficient) AND variation (e.g. standard deviation) or associated estimates of uncertainty (e.g. confidence intervals) |
| ☐ | ☒ | For null hypothesis testing, the test statistic (e.g. *F*, *t*, *r*) with confidence intervals, effect sizes, degrees of freedom and *P* value noted<br>*Give P values as exact values whenever suitable.* |
| ☒ | ☐ | For Bayesian analysis, information on the choice of priors and Markov chain Monte Carlo settings |
| ☐ | ☒ | For hierarchical and complex designs, identification of the appropriate level for tests and full reporting of outcomes |
| ☐ | ☒ | Estimates of effect sizes (e.g. Cohen's *d*, Pearson's *r*), indicating how they were calculated |

*Our web collection on statistics for biologists contains articles on many of the points above.*

## Software and code

Policy information about availability of computer code

| | |
|---|---|
| Data collection | Thermo Xcalibur (4.0.27.19), LTQ Tune plus (2.5.5 SP2), MaxQuant Live (Version 1.1) |
| Data analysis | MaxQuant (1.6.2.10), Spectronaut (13.12.200217.43655), Perseus (1.6.7.0), STRING app (1.5.1) in Cytoscape (3.7.2), Python (3.7.7), pantherdb website (http://pantherdb.org/), R (3.6.2), numpy (1.18.1), pandas (0.24.2) pyteomics (4.2), HoloViz (0.11.3), holoviews (1.13.2), bokeh (2.0.1), plotly (4.6.0), matplotlib (3.0.3), CFX Manager Software (Version 3.1) |

For manuscripts utilizing custom algorithms or software that are central to the research but not yet described in published literature, software must be made available to editors and reviewers. We strongly encourage code deposition in a community repository (e.g. GitHub). See the Nature Research guidelines for submitting code & software for further information.

## Data

Policy information about availability of data

All manuscripts must include a data availability statement. This statement should provide the following information, where applicable:

- Accession codes, unique identifiers, or web links for publicly available datasets
- A list of figures that have associated raw data
- A description of any restrictions on data availability

All mass spectrometry data have been deposited on the ProteomeXchange Consortium via the PRIDE database with the dataset identifier PXD019854. A file linking mass spectrometry raw data in the ProteomeXchange folder to the associated experiments in the manuscript is available (Supplementary Data 8). The proximity analysis tool for the investigation of cycling diGly sites is available on http://cyclingubi.biochem.mpg.de. Information about protein domains was obtained from UniProt (https://www.uniprot.org/, accessed 25.05.2020). Source data are provided with this paper. Custom code for the proximity analysis, implemented on http://cyclingubi.biochem.mpg.de has been deposited on GitHub (https://github.com/ibludau/CyclingProximityAnalysis)

# Field-specific reporting

Please select the one below that is the best fit for your research. If you are not sure, read the appropriate sections before making your selection.

[x] Life sciences [ ] Behavioural & social sciences [ ] Ecological, evolutionary & environmental sciences

For a reference copy of the document with all sections, see nature.com/documents/nr-reporting-summary-flat.pdf

# Life sciences study design

All studies must disclose on these points even when the disclosure is negative.

| | |
|---|---|
| Sample size | No sample size calculation was performed. For DIA library construction samples were measured in singlicates, as the purpose was only to identify diGly sites, not quantify them. Titration experiments were acquires in singlicates or duplicates as this is sufficient to estimate the general trends. Experiments comparing DIA with DDA were performed in workflow triplicates and each sample was measured four times (2XDIA and 2XDDA). This resulted in six measurements per condition allowing robust statistical analysis. In dilution experiments, three technical triplicates were done for each acqusition strategy, allowing for statistical analysis. In the TNF signaling experiment, each condition (treated or untreated) was measured in three workflow replicates, each was measured in analytical duplicates (a total of six replicates per condition). For the circadian cycle experiment, four biological replicates were measured per time point. |
| Data exclusions | No data was excluded |
| Replication | Three workflow replicates, each measured in analytical duplicates, were acquired for TNF signaling experiment and DIA-DDA comparisons. Analytical replicates describe repetitive LC-MS/MS measurements, picked the sample from the same vial/tube. Workflow replicates describe LC-MS/MS measurements that were individually processed from the diGly-peptide enrichment step onward. Four biological replicates for the circadian cycle experiments were performed, harvesting cells from separate plates and processing them individually. Reproducibility between biological replicates was confirmed by Pearson correlation coefficients. Western-blots were replicated twice with similar results obtained. |
| Randomization | Titration and dilution experiments were measured from low to high concentrations to avoid carryover effect. Analytical replicates of DDA and DIA were measured in alternating order (DDA, DIA, DDA, DIA). Workflow replicates for TNF signaling and DDA/DIA comparison experiments were measured in alternating order (treated, untreated, treated, untreated, treated, untreated). Each workflow replicate was measured twice via DIA and twice via DDA in alternating order (DDA, DIA, DDA, DIA). In the circadian experiment, all time points of one biological replicate were batched and the order of time point measurements was reversed between replicates (Replicate 1 - 0,4,8,12,16,20,24,28,32; Replicate 2 - 32,28,24,20,16,12,8,4,0; Replicate 3 - 0,4,8,12,16,20,24,28,32; Replicate 4 - 32,28,24,20,16,12,8,4,0). |
| Blinding | Blinding was not carried out. The main objective was the technical evaluation of DIA method in ubiquitinome analysis. |

# Reporting for specific materials, systems and methods

We require information from authors about some types of materials, experimental systems and methods used in many studies. Here, indicate whether each material, system or method listed is relevant to your study. If you are not sure if a list item applies to your research, read the appropriate section before selecting a response.

## Materials & experimental systems

| n/a | Involved in the study | |
|---|---|---|
| [ ] | [x] | Antibodies |
| [ ] | [x] | Eukaryotic cell lines |
| [x] | [ ] | Palaeontology and archaeology |
| [x] | [ ] | Animals and other organisms |
| [x] | [ ] | Human research participants |
| [x] | [ ] | Clinical data |
| [x] | [ ] | Dual use research of concern |

## Methods

| n/a | Involved in the study | |
|---|---|---|
| [x] | [ ] | ChIP-seq |
| [x] | [ ] | Flow cytometry |
| [x] | [ ] | MRI-based neuroimaging |

## Antibodies

Antibodies used

Phospho-NF-κB p65 (Ser536) (93H1) Rabbit mAb, CST, 3033P, Lot6
NF-κB p65 (C22B4) Rabbit mAb, CST, 4764P, Lot3
IκBα Antibody, CST, 9242, Lot10
p38 MAPK Antibody, CST, 9212, Lot26
Phospho-p38 MAPK (Thr180/Tyr182) (3D7) Rabbit mAb, CST, 9215, Lot7
β-Actin (13E5) Rabbit mAb, CST, 4970, Lot11
anti RIP, BD Bioscience, 610458, Lot6230745
anti TRAF2, CST, 4712. Lot2
anti β-actin, Santa Cruz, sc-47778, Lot D1520

| | |
|---|---|
| | PTMScan® Ubiquitin Remnant Motif (K-ε-GG) Kit, Lot 23, CST, 5562 (used as described in manuscript) |
| Validation | Validation performed by detection of K-GG peptides after enrichment by LC-MS/MS, also see PTMScan® Ubiquitin Remnant Motif (K-ε-GG) Kit: https://www.cellsignal.com/products/proteomic-analysis-products/ubiquitin-remnant-motif-k-e-gg-kit/5562?site-search-type=Products&N=4294956287&Ntt=5562+&fromPage=plp&_requestid=907690<br>TRAF2 antibody: https://www.cellsignal.de/products/primary-antibodies/traf2-antibody/4712<br>RIPK1 antibody: https://www.bdbiosciences.com/us/applications/research/apoptosis/purified-antibodies/purified-mouse-anti-rip-38rip/p/610458<br>Actin antibody (UBA pulldown): https://www.scbt.com/p/beta-actin-antibody-c4<br>Actin antibody: https://www.cellsignal.de/products/primary-antibodies/b-actin-antibody/4967<br>p65 antibody: https://www.cellsignal.de/products/primary-antibodies/nf-kb-p65-c22b4-rabbit-mab/4764<br>Phospho p65 antibody: https://www.cellsignal.com/products/primary-antibodies/phospho-nf-kb-p65-ser536-93h1-rabbit-mab/3033<br>IkBa antibody: https://www.cellsignal.de/products/primary-antibodies/ikba-antibody/9242<br>Phospho p38 antibody: https://www.cellsignal.de/products/primary-antibodies/phospho-p38-mapk-thr180-tyr182-3d7-rabbit-mab/9215<br>p38 antibody: https://www.cellsignal.de/products/primary-antibodies/p38-mapk-antibody/9212 |

## Eukaryotic cell lines

Policy information about cell lines

| | |
|---|---|
| Cell line source(s) | U-2 OS, ATCC, HTB-96 ; HEK 293T, DSMZ, ACC 635 |
| Authentication | No authenticated performed |
| Mycoplasma contamination | Cell lines were tested mycoplasma contamination |
| Commonly misidentified lines (See ICLAC register) | None |
